# Highly Efficient Stable Expression of Indoleamine 2,3 Dioxygenase Gene in Primary Fibroblasts

**DOI:** 10.1007/s12575-010-9028-6

**Published:** 2010-03-27

**Authors:** Alireza Moeen Rezakhanlou, Darya Habibi, Amy Lai, Reza B Jalili, Christopher J Ong, Aziz Ghahary

**Affiliations:** 1 Burn and Wound Healing Research Group, Department of Surgery, University of British Columbia, Vancouver, BC, Canada; 2 The Prostate Centre at Vancouver General Hospital, Department of Surgery, University of British Columbia, Vancouver, BC, Canada; 3 The Endocrinology and Metabolism Research Centre, University of Tehran/Medical Sciences, Tehran, Iran; 4 Burn and Wound Healing Research Lab, Rm 350, Jack Bell Research Centre, 2660 Oak Street, Vancouver, BC, Canada, V6H 3Z6

**Keywords:** Lentiviral vector, Indoleamine 2, 3 dioxygenase, Primary fibroblast, Transplantation, Immunogenicity

## Abstract

Indoleamine 2,3 dioxygenase (IDO) is a potent immunomodulatory enzyme that has recently attracted significant attention for its potential application as an inducer of immunotolerance in transplantation. We have previously demonstrated that a collagen matrix populated with IDO-expressing fibroblasts can be applied successfully in suppressing islet allogeneic immune response. Meanwhile, a critical aspect of such immunological intervention relies largely on effective long-term expression of the IDO gene. Moreover, gene manipulation of primary cells is known to be challenging due to unsatisfactory expression of the exogenous gene. In this study, a lentiviral gene delivery system has been employed to transduce primary fibroblasts. We used polybrene to efficiently deliver the IDO gene into primary fibroblasts and showed a significant increase (about tenfold) in the rate of gene transfection. In addition, by the use of fluorescence-activated cell sorting, a 95% pure population of IDO-expressing fibroblasts was successfully obtained. The efficiency of the IDO expression and the activity of the enzyme have been confirmed by Western blotting, fluorescence-activated cell sorting analysis, and Kynurenine assay, respectively. The findings of this study revealed simple and effective strategies through which an efficient and stable expression of IDO can be achieved for primary cells which, in turn, significantly improves its potential as a tool for achieving immunotolerance in different types of transplantation.

## 1 Introduction

Indoleamine 2,3-dioxygenase (IDO) is a monomeric, heme-containing enzyme that catalyzes the rate limiting step of conversion of tryptophan to kynurenine [1]. Recently, tryptophan catabolism has been implicated in immunological tolerance. One theory proposes that degradation of tryptophan suppresses T cell proliferation by reducing the availability of this essential amino acid in local tissue environments, thereby sensitizing T cells to apoptosis [2]. Another theory suggests that the major tryptophan metabolite, kynurenine, suppresses immune reactivity through direct interaction with effector T lymphocytes [3]. The immunomodulatory effects of tryptophan deficiency and excess kynurenine caused by IDO are of particular interest in the field of transplantation [4].

A critical aspect of an immunological intervention using IDO is the requirement to achieve effective expression of this gene. However, genetic manipulation by non-viral transfection approaches has been challenging due to the issues of low transfection efficiency, loss of cell viability, and difficulty in obtaining stable transfection [5,6]. Previously, we have shown that by using dermal fibroblasts transduced with an IDO-expressing adenoviral vector, IDO functions as a local immunosuppressive factor [7], and local expression of IDO suppresses islet allogeneic immune response in mouse islet transplantation [8]. Furthermore, we have used the local immunosuppressive effect of IDO in the development of a non-rejectable skin substitute [9]. These findings demonstrate that IDO has considerable potential for immunoregulation and induction of immunotolerance in transplantation.

Nevertheless, the immunogenicity and transient gene expression of adenoviral vectors may hinder its clinical use in transplantation. Thus, in order to sustain the efficacy of IDO activity in the local environment of transplanted organ, it is essential to prolong the expression of functional IDO protein. In fact, it was shown that lentiviral vectors can maintain efficient long-term target gene expression in vivo for more than 4 years [10]. It is has been reported that polybrene can markedly enhance the retrovirus transduction efficiency [11]. In this report, we constructed an IDO-expressing lentiviral vector and showed that treatment of cells with polybrene-enhanced IDO transduction efficiency almost ten times. To enrich IDO-expressing cells and thereby optimize transplantation immunotolerance, we selectively isolated the IDO-positive cells by fluorescence-activated cell sorting (FACS) and obtained a greater than 95% pure population of IDO-expressing cells.

## 2 Materials and Methods

### 2.1 Plasmid Constructions

A lentiviral construct for expressing the IDO gene was generated using the pLC-E vector [12] modified from the lentiviral backbone FUGW [13]. For visualization of the lentiviral-mediated IDO expression, a sequence encoding the red fluorescent mCherry protein under the control of the UbC promoter was incorporated into the vector. The human IDO gene (NM_002164; a generous gift from Dr. JM Carlin of Miami University) was generated by PCR using a full-length cDNA encoding the gene as template and the forward primer (5'-GGGGACAAGTTTGTACAAAAAAGCAGGCT TCACCATGGCACACGCTATGGAAAACTCCTGG-3') and reverse primer (5'-GGG GACCACTTTGTACAAGAAAGCTGGGTCCTAACCTTCCTTCAAAAGGGATTTCTC-3'). The amplified PCR product was first inserted into an entry vector (pDON201) and then Gateway (Invitrogen) cloned into a lentiviral pLC-E expression vector. The IDO gene is expressed under the control of EF1-α promoter, and the mCherry red fluorescent gene, a reporter gene, is expressed under the control of a ubiquitin promoter. The plasmid was amplified in competent DH10-B bacteria and purified using the Qiagen Plasmid DNA Maxi-prep kit (Qiagen). Sequence of the IDO/pLC-E construct was confirmed by DNA sequencing analysis.

### 2.2 Cell Culture

Skin samples were collected from 6- to 8-week-old male C57BL/6 (B6) mice according to the guidelines of the Animal Policy and Welfare Committee of the University of British Columbia. The samples were then washed in sterile dulbecco's modified eagle medium (DMEM) (Invitrogen Life Technologies, Carlsbad, CA) supplemented with antibiotic–antimycotic preparation [100 U/ml penicillin, 100 mg/ml streptomycin, 0.25 mg/ml amphotericin B (Invitrogen)]. Cultures of fibroblasts were established as previously described [14] and grown in DMEM supplemented with 10% fetal bovine serum (FBS; Invitrogen Life Technologies, Carlsbad, CA). Confluent cells were released by trypsinization, reseeded onto 75 cm^2^ cell culture flasks (BD Biosciences, MA), and incubated in a humidified incubator at 37°C supplied with 5% CO^2^. Fibroblasts at passages three to five were used in all experiments.

#### 2.2.1 Lentiviral Vector Production and Cell Transduction

Replication-defective lentiviral vectors were generated as previously described [15,16] by transient transfection of 293T human kidney cells with the IDO/pLC-E vector, R8.9 (packaging plasmid), and VSV-G (envelope plasmid). Some 293T cells were seeded the night before transfection in DMEM medium supplemented with 10% fetal bovine serum (Invitrogen Life Technologies, Carlsbad, CA). The cells were replaced with DMEM+10% FBS and transfected at 60–70% confluency using ProFectin (Mammalian Transfection System Calcium Phosphate; Promega). For each 100-mm^2^ round plate, 10 μg IDO/pLC-E vector, 7.5 μg R8.9 plasmid, and 2.5 μg VSV-G plasmid were used. The culture medium was replaced with a fresh medium containing DMEM and 5% FBS 12–16 h after transfection. Supernatant was harvested 30 h after the medium change. The vector stocks were concentrated by centrifugation at 126,000*g* for 90 min using Beckman Ultracentrifuge and stored at -80°C until use.

For transduction, 293T cells or mouse fibroblasts were seeded on flat-bottom 6-well cell culture plates (Corning Incorporated, Corning, NY, USA) and incubated with the same titer of IDO-lentiviral vector for 24 h in the absence or presence of 10 ug/ml polybrene. Cell supernatant containing lentiviral vectors were removed from the culture 30 h later.

### 2.3 SDS-PAGE and Western Blotting

For detection of the IDO protein expression, transduced cells were harvested 24 h post-transduction and washed twice with PBS. Cells were then lysed in lysis buffer (50 mM Tris-HCl, pH 7.4; 10 mM EDTA; 5 mM EGTA; 0.5% NP40; 1% Triton X-100; and protease inhibitor cocktail (Sigma). Equal amounts of total protein from each individual 293T cell culture were separated by 10% sodium dodecyl sulfate - polyacrylamide gel electrophoresis (SDS-PAGE). Proteins were then transferred to a PVDF membrane (Millipore Corp., Bedford, MA) and immunoblotted with a polyclonal anti-human IDO antibody (Washington Biotechnology Inc., Baltimore, MD) at final dilution of 1:5,000. Horseradish peroxidase-conjugated goat anti-rabbit IgG was used as the secondary antibody for the enhanced chemiluminescence detection system (Amersham Biosciences, UK). Blots were then stripped and reprobed for β-actin as a control for protein loading.

### 2.4 Fluorescence Microscopy

The lentiviral-mediated IDO expression in transduced 293T cells, and mouse fibroblasts were examined by fluorescence microscopy with a Zeiss Axiovert 200 M microscope. Images from identical areas of cultured cells were recorded using both fluorescence and bright-field microscopy. Images were captured using Northern Eclipse image analysis software.

### 2.5 Kynurenine Assay

The biological activity of IDO was evaluated by measuring the level of tryptophan degradation product, L-kynurenine, present in the conditioned medium of transduced cells. The amount of L-kynurenine was measured by a previously established method [17]. Proteins in the conditioned medium were precipitated by trichloroacetic acid. After centrifugation, 0.5 ml of supernatant was incubated with an equal volume of Ehrlich's reagent at room temperature for 10 min. The reaction mixture was measured spectrophotometrically at 490 nm. The concentration of kynurenine in the conditioned medium was calculated according to a standard curve of defined kynurenine concentration (0–20 mg/ml).

### 2.6 Flow Cytometry

To determine the transduction efficiency of the IDO-lentiviral vector, and in order to sort mCherry-positive cells, non-transduced cultured fibroblasts, fibroblasts transduced with IDO-lentiviral vector in the absence or presence of polybrene were trypsinized, collected, and centrifuged. The cell pellet was washed twice with PBS and resuspended at 10^6^ cells/mL for flow cytometry and at 10^7^ cells/mL for sorting in PBS±2% FBS. For flow cytometry, BD LSRII with 630 LP and 670/30 detectors were used. For sorting, BD FACSAria with a blue laser detector was used. In each set, live cells were gated using forward scatter channel vs side scatter channel followed by gating on the IDO-expressing mCherry-positive cells. The number of mCherry-positive cells for fibroblasts transduced with IDO-lentiviral vector in the presence of polybrene after sorting was also determined by flow cytometry analysis. The average purity was greater than 95%.

## 3 Results and Discussion

### 3.1 Characterization of Lentiviral-Mediated IDO Expression

**Figure 1:**
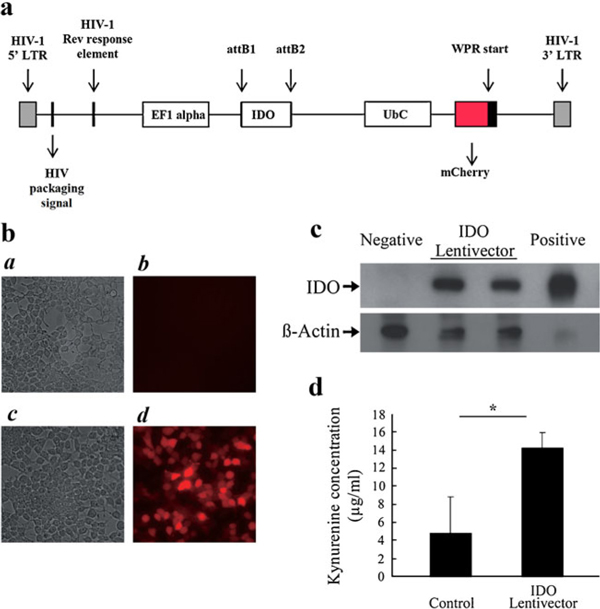
**Construction of a lentiviral-based vector for delivering the IDO gene**. **a** Schematic diagram of the IDO–mCherry lentiviral construct. **b** Fluorescence microscopy analysis of IDO-expressing cells. Panels *a* and *b* as well as *c* and *d* represent bright-field and fluorescent images of the IDO–mCherry (*red*) expression in non-transduced and transduced 293T cells, respectively. **c** Lentiviral vector-mediated IDO protein expression in transduced 293T cells. The *arrow* on the *upper band* shows the IDO protein. The *IDO-lentiviral vector lanes* represent two separate viral preparations. The blot was reprobed with a β-actin antibody as a loading control. **d** Kynurenine assay. Functional IDO activity was evaluated by measuring the content of kynurenine in the conditioned media of non-transduced and IDO-lentiviral vector-transduced cells. The data shown are the mean and standard deviation of kynurenine in conditioned media of three separate experiments.

Schematic diagram of the IDO–mCherry lentiviral vector is shown in Figure 1a. IDO-expressing lentiviral vector preparations were generated. To examine the efficacy of the generated lentiviral vector, the vector was first collected from the transfected 293T cells and used to transduce a fresh culture of 293T cells. The red fluorescent mCherry protein acts as a reporter for the promoter activity of the IDO gene, verifying that the IDO gene is present and expressed in the transduced cells (Figure 1b). As shown in Figure 1b*d*, majority of transduced cells express red fluorescent mCherry protein as a reporter gene for IDO expression compared with that of control cells (Figure 1b*b*). Figure 1b panels *c* and *a* show the images of the same cells in bright-field. Furthermore, to examine the protein expression of the exogenously introduced IDO gene, lysates of the transduced cells were subjected to SDS-PAGE and immunoblotted with an anti-IDO polyclonal antibody (Figure 1c). As seen in Figure 1c, the IDO expression was positive in both viral preparations and the transduction was successful. Non-transduced cell lysate was used as a negative control and recombinant IDO protein was used as a positive control. In order to test whether the overexpressed IDO protein was functional, kynurenine assay was performed to measure the concentration of the major tryptophan degradation product (L-kynurenine) in the conditioned media. In comparison with non-transduced cells, the concentration of L-kynurenine in the conditioned media of transduced cells increased nearly threefold (Figure 1d) and the result was significantly different from that of non-transduced cells (*P* < 0.02, *n* = 3). The results show that the IDO-expressing lentiviral vector generated by transient transfection of 293T cells was functional and suitable for transduction of primary fibroblasts.

### 3.2 Transduction of Primary Mouse Fibroblasts with IDO-Expressing Lentiviral Vector

**Figure 2:**
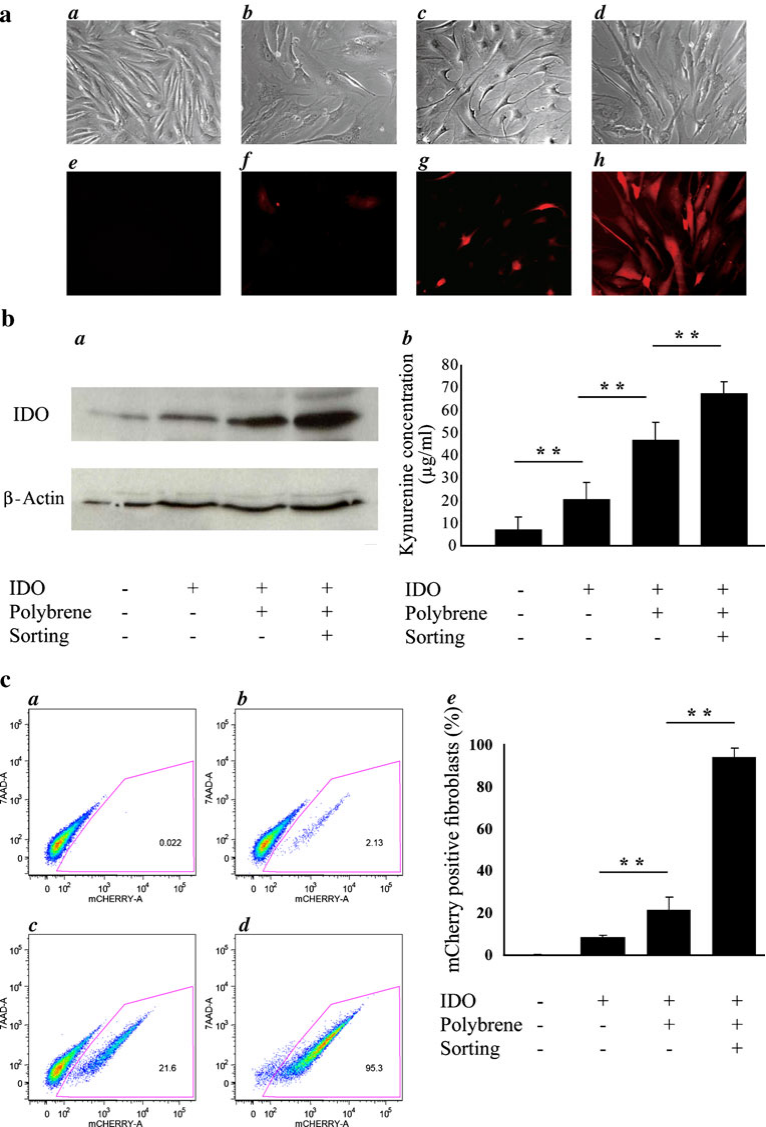
**Transduction of mouse fibroblasts with IDO-expressing lentiviral vector**. **a** Fluorescence microscopy analysis of IDO-lentiviral vector-transduced fibroblasts. Cultured mouse fibroblasts were incubated with IDO-lentiviral vector either in the presence or absence of polybrene. Cells that were transduced in the presence of polybrene were then sorted by FACS. Panels *a* and *e*, *b* and *f*, *c* and *g*, as well as *d* and *h* represent bright-field and fluorescence images of non-transduced fibroblasts, IDO-lentiviral vector-transduced fibroblasts, fibroblasts transduced with IDO-expressing lentiviral vector in the presence of polybrene, and fibroblasts transduced with IDO-expressing lentiviral vector in the presence of polybrene after sorting, respectively. **b** These *panels* show the Western blot analysis (*a*) as well as kynurenine levels (*b*) in different indicated cells. The data shown are the mean and standard deviation of kynurenine measurements obtained from three different experiments. **c** Flow cytometry analysis of the IDO-expressing cell population. Fibroblasts were left either non-transduced or transduced with IDO-expressing lentiviral vector in the absence or presence of polybrene and flow cytometry was then conducted by gating the cells based on their mCherry fluorescence. The selected cell population was prepared by cell sorting and the number of mCherry-positive cells was greater than 95%. Panel *a* shows non-transduced fibroblasts, *b* transduced IDO-expressing fibroblasts in the absence of polybrene, *c* transduced IDO-expressing fibroblasts in the presence of polybrene, and *d* FACS-sorted IDO-expressing fibroblasts (in the presence of polybrene). Panel *e* shows the percentage of mCherry-positive fluorescent cells in the total cell population studied as detected by flow cytometry. The data shown are the mean and standard deviation of mCherry-positive cells obtained from three separate experiments.

Dermal fibroblasts were transduced with the IDO-expressing lentiviral vector. To analyze lentiviral-mediated IDO expression, transduced cells were visualized by fluorescence microscopy 24 h post-transduction (Figure 2a). The Figure 2a upper and lower panels are bright-field images and fluorescence images, respectively. Fluorescence images are images of the same groups of cells captured by the bright-field microscope, and the fluorescence is due to expression of the mCherry gene. Figure 2a panels *a* and *e* as well as *b* and *f* represent non-transduced fibroblasts and IDO-lentiviral vector-transduced fibroblasts, respectively. To facilitate delivery of the IDO-lentiviral vector, polybrene was added to cells at the time of transduction. The cells were similarly examined by fluorescence microscopy 48 h post-transduction (Figure 2a, panels *c* and *g*). Transduced fibroblasts in the presence of polybrene were gated and sorted by FACS and examined by fluorescence microscopy (Figure 2a, panels *d* and *h*). As the fluorescent images show, the level of mCherry-positive cells increased in the presence of polybrene and after sorting. The expression of IDO protein in these cells was determined by Western blot analysis (Figure 2b, panel *a*). IDO protein level increased in the presence of polybrene and after sorting. The functionality of the overexpressed IDO protein was assessed by kynurenine assay (Figure 2b, panel *b*). The kynurenine content in the conditioned media of mouse fibroblasts transduced in the presence of polybrene increased approximately twofold when compared with that of control with no polybrene treatment (*P* < 0.01, *n* = 3). This finding revealed that polybrene significantly enhanced the transduction efficiency in primary mouse fibroblasts as well as the IDO protein and its enzymatic activity.

#### 3.2.1 Analysis of Improvement of Lentiviral Vector Transduction by Flow Cytometry

To maximize the chances for successful transplantation and reduce the risk of immunorejection, it would be advantageous to have a highly pure population of IDO-expressing cells. By FACS, we sorted the IDO–mCherry fluorescent protein-expressing cells (Figure 2c). Panel *a* of Figure 2c shows that there is less than 0.02% of autofluorescent cells in the negative-cell population. As shown in panels *b* and *c* of Figure 2c, treatment of IDO-lentiviral vector-transduced cells by polybrene increased the number of mCherry-positive cells by tenfold. These cells were then gated and sorted by FACS. Figure 2c, panel *d*, shows that more than 95% of the cell population after sorting were expressing IDO, and this was confirmed by flow cytometry, fluorescence microscopy, and kynurenine assay. The transduction efficiency was shown as percentage of mCherry-positive fluorescent cells in the total cell population studied (Figure 2c, panel *e*). Transduction of mouse fibroblasts in the presence of polybrene showed a significant increase in the number of IDO-expressing cells (*P* ≤ 0.01, *n* = 3). As a result, we successfully obtained a cell population that contains more than 95% IDO-expressing primary fibroblasts (Figure 2c, panels *d*–*e*).

As efficient gene delivery is a prerequisite for successful gene therapy, we believe that polybrene significantly increases lentiviral-mediated delivery of the IDO gene into target cells and that should make this enzyme a promising and highly attractive strategy for improving the outcome of cell and possibly organ transplantation.

## Competing interest statement

The authors declare that they have no competing interests.
